# Effect of Radiofrequency Electromagnetic Fields on Thermal Sensitivity in the Rat

**DOI:** 10.3390/ijerph17207563

**Published:** 2020-10-18

**Authors:** Nihal S. Ouadah, Kelly Blazy, Anne-Sophie Villégier

**Affiliations:** 1 Unité de Toxicologie Expérimentale, Institut National de l’Environnement Industriel et des Risques, 60550, Verneuil-en-Halatte, France; nihal75@live.fr (N.S.O.); kellyblazy@free.fr (K.B.); 2 Unité mixte PERITOX UMI-01 INERIS CHU Amiens-Picardie Hôpital Sud, 80480 Salouël, France

**Keywords:** electromagnetic fields, radiofrequency, thermal preference, nociception, restraint, stress-induced analgesia

## Abstract

The World Health Organization and the French Health Safety Agency (ANSES) recognize that the expressed pain and suffering of electromagnetic field hypersensitivity syndrome (EHS) people are a lived reality requiring daily life adaptations to cope. Mechanisms involving glutamatergic N-methyl d-aspartate (NMDA) receptors were not explored yet, despite their possible role in hypersensitivity to chemicals. Here, we hypothesized that radiofrequency electromagnetic field (RF-EMF) exposures may affect pain perception under a modulatory role played by the NMDA receptor. The rats were exposed to RF-EMF for four weeks (five times a week, at 0 (sham), 1.5 or 6 W/kg in restraint) or were cage controls (CC). Once a week, they received an NMDA or saline injection before being scored for their preference between two plates in the two-temperatures choice test: 50 °C (thermal nociception) versus 28 °C. Results in the CC and the sham rats indicated that latency to escape from heat was significantly reduced by −45% after NMDA, compared to saline treatment. Heat avoidance was significantly increased by +40% in the 6 W/kg, compared to the sham exposed groups. RF-EMF effect was abolished after NMDA treatment. In conclusion, heat avoidance was higher after high brain-averaged specific absorption rate, affording further support for possible effect of RF-EMF on pain perception. Further studies need to be performed to confirm these data.

## 1. Introduction

The World Health Organization and the French Agency for Food, Environmental and Occupational Health and Safety (ANSES) recognize that the expressed pain and suffering of electromagnetic field hypersensitivity syndrome (EHS) people are a lived reality requiring daily life adaptations to cope [1].

EHS people connect non-specific symptoms to electromagnetic field (EMF) exposures, such as pain in the form of headaches, dizziness, burning sensations or ache in muscles [2,3]. They mainly incriminate relay antennas and individual wireless equipment emitting radio frequency (RF) waves, such as cell phones, tablets or WIFI. The equipment has appeared over the past thirty years and were initially purchased by a minority of households. They subsequently spread to the entire population. In France, in 2018, equipment rate for mobile phones reached 95.4% of households [4]. In 2017, indoor and outdoor environmental measures indicated mobile telephony was the largest contributor, and the 900 MHz band, the most represented mobile telephony frequency band [5]. For local exposures to the brain in the general population at 900 MHz, the International Commission on Non-Ionizing Radiation Protection (ICNIRP) guideline is 2 W/kg specific absorption rate (SAR) averaged over any 10 g of contiguous tissue [6]. People exposure to a second generation cell phone emitting at 900 MHz is about 3.4 Volts/m at 50 cm [7]. Environmental exposure from relay antennas is lower due to their distance to people habitations.

The prevalence of EHS reaches around 5% (between 1.2% and 8.8%) but remains very difficult to evaluate in France and worldwide. The ANSES report highlighted the hypothesis of possible RF-EMF effects to the brain, on the production of neurotransmitters, and the lack of animal model for the study of EHS [1]. In that sense, the ANSES expert panel recommends French governmental funding for fundamental research on EHS and EMF health effects.

In 2008, Landgrebe et al. proposed that the EHS could belong to and share common etiologic mechanisms with the functional somatic syndromes, such as multiple chemical sensitivity (MCS) [8]. Both the EHS and the MCS share pain symptoms and suspected environmental causes. MCS was linked with an excitability of certain areas of pain perception in the case of chemical exposures. An increase in the glutamatergic N-methyl d-aspartate (NMDA) receptor activity was shown with seven categories of chemical agents known to trigger MCS. The NMDA receptor increases both nitric oxide and peroxynitrite levels, and plays a role in neurogenic inflammation and peripheral sensitivity. The resulting toxic responses were mitigated by the action of NMDA antagonists [9,10,11,12]. Despite the possible role played by NMDA receptors in functional somatic syndromes, the mechanisms involving NMDA receptors were not explored yet in the case of EHS.

In previous data in healthy volunteers, cutaneous thermal pain perception was influenced by an overtime effect of head RF-EMF exposure (UMTS 1947 MHz, 1.75 W/kg) using visual analogue subjective pain rating scale [13]. In rodents, Mathur et al. [14] showed that intermittent RF-EMF exposures (0.4 W/kg, 73.5 MHz, whole body) heightened the emotional component of phasic pain. Bodera et al. [15] showed that RF-EMF exposures (1500 MHz and 1800 MHz) transiently suppressed the effect of tramadol, an analgesic acting through opioids and monoaminergic transmissions. On the contrary, in snails, Nittby et al. [16] showed analgesia after exposure (Global System for Mobile Communications (GSM), 1900 MHz, 48 mW/kg). In the same way, Maillefer and Quock [17] showed analgesia in mice after exposure to continuous-wave (2.45 GHz, 20 mW/cm^2^, 46 W/kg) using the acetic acid abdominal constriction test. This effect was reversed by naltrexone, an opioid receptor antagonist.

Our previous work suggested that high brain averaged (BA) SAR RF-EMF exposures could impact pain memory, plasmatic corticosterone, and astrocyte activation [18,19], i.e., variables which may influence pain perception [20,21]. Here, our first hypothesis was that high BASAR RF-EMF exposures may impact thermal nociception. Our second hypothesis was that this effect may occur under a modulatory role played by the NMDA receptor.

## 2. Materials and Methods

### 2.1. Animals

The protocols were approved by the French State Council guidelines for the care and use of laboratory animals (Decree n° 87-849, 19 October 1987). At post-natal day (P) 28 (90–110 g), 71 male Wistar rats (Janvier Lab, St Berthevin, France) were housed (two per cage) in a controlled environment (room temperature 22 °C, 12 h light/dark inverted circadian cycle, food and water ad libitum). Animals were handled daily for one week to acclimatize before the start of experiment. Cages were enriched with plastic cylinders identical in shape to RF-EMF exposure rockets.

### 2.2. Experimental Groups

**Figure 1:**
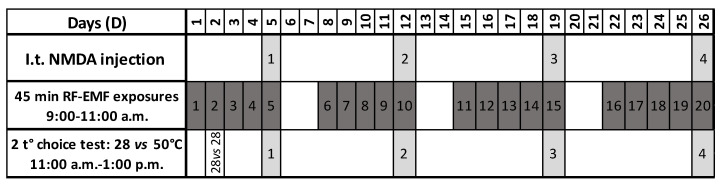
Rats underwent 20 radiofrequency electromagnetic field (RF-EMF) exposures (0, 1.5 or 6 W/kg) between 9:00 and 11:00 a.m. On day 2, side preference was measured in the test apparatus (28 °C versus 28 °C). The 5th, 10th, 15th and 20th RF-EMF exposures were performed immediately after the i.t. injections of N-methyl d-aspartate (NMDA) or saline, followed by the two-temperatures choice test (28 °C versus 50 °C, between 11:00 a.m. and 1:00 p.m.).

Rats were randomly assigned to independent RF-EMF exposure groups: to 0 (sham), 1.5 or 6 W/kg. A total of 71 rats underwent five daily RF-EMF exposures per week for four weeks (on days (D)1–5, 8–12, 15–19 and 22–26) (Figure 1). During each exposure session, both the sham and the RF-EMF exposed groups spent 45 min in the restrainers. The RF-EMF exposure system was switched on only for the exposed groups but not for the sham groups. On D2, the side preference of each rat was measured in the temperature choice test apparatus. Each rat was tested four times using the two-temperatures choice test (28 °C versus 50 °C) on D5, D12, D19 and D26. In these sessions, RF-EMF or sham exposures were performed immediately after the intrathecal (i.t.) injections of NMDA or saline and were followed by the two-temperatures choice test (*n* = 6–12/group). A cage control group (CC *n* = 6–8, no restraint) was used to validate the test. The CC groups were not placed in the restrainers but underwent the 4 i.t. NMDA or saline injections and the temperatures choice tests. We cannot exclude that the repetition of the whole protocol (NMDA i.t. administrations, restraint, heat nociceptive stimulation and/or RF-EMF exposures) may have influenced data variability through possible side effects [17,18,22,23,24,25].

### 2.3. RF-EMF Exposures

A radiofrequency power source (900-64 type, generator RFPA SA, RFS9001800-28 model, Radiofrequency Power Amplifier, Artigues-près-Bordeaux, France) emitted a GSM 900 MHz EMF (1/8 duty factor) pulse modulated at 217 Hz. Its output was connected to a loop antenna allowing head exposure of animals under restraint in Plexiglas rockets in Faraday cages. One loop antenna was connected to a detector to control incident and reflected powers. The restrainers consisted of a truncated cone into which the rat’s head was inserted, and a cylinder (4.5, 5 and 6 cm in diameters to adapt to rat body size growth between P30 and P60). A Plexiglas disk was placed at the back to prevent the rat from backing out of the rocket. Dosimetry in the brain was performed numerically and experimentally [26]. With an input power of 1 W, BASAR was 6 W/kg, and maximum SAR inside the skull was estimated at 15.5 ± 5 W/kg. Numerically, finite difference time domain calculations were done on homogeneous and non-homogeneous phantoms. The agreement among these independent methods was very good. SAR was calculated as followed: SAR = Cp·Δ*T*/dt and SAR = σ|E^2^|/ρ (Cp: tissue’s calorific capacity in J/kg·°C; Δ*T*, temperature variation in °C; dt, time variation in s; σ, conductivity in S/m; *E*, electric field in V/m; *ρ*, density in kg/m^3^).

### 2.4. I.T. NMDA Injections

The rats were placed at 37 °C and anesthetized (isoflurane 3.5%). NMDA (0 or 5 μg/kg in 5 µL saline) was injected using a 28 Gauge 1” needle connected to a 28 µL Hamilton syringe, inserted between the dorsal facets of L5 and L6 vertebra. NMDA at high doses may induce epileptic crises. Here, NMDA dose was under the epileptic threshold. The injection was performed 50 min prior to the behavioral tests to allow the rat to perform the operant response right following the combined treatment NMDA with RF-EMF exposures.

### 2.5. Two-Temperatures Choice Test

The test apparatus consisted of two contiguous metal plates surrounded by a plastic enclosure (Bioseb, France) [27]. The side preference was tested by measuring the rat’s exploration of the two plates set at 28 °C (neutral temperature) for 3 min. The rats had no preference for one side when both plates were set at neutral temperature (Δ_28–28 °C_ = 0 ± 12, data not shown). During the tests, the rats were placed at the intersection of the two plates (two paws (hind and back) on each side), one set at 28 °C, the other at 50 °C (hot temperature) and explored for 3 min. Plates were wiped with a 10% ethanol solution and their temperatures were permuted between tests. The time spent on each plate, the latency to escape the hot plate and the number of crossings between the plates (locomotor activity) were measured by a computer connected to an infrared camera. Heat avoidance was expressed as the delta (Δ_28–50 °C_) between the times spent at 28 °C and at 50 °C.

### 2.6. Statistics

Data were presented as means ± standard error of the mean and were analyzed using SPSS Statistics 19 software (IBM, Chicago, IL, USA). Effects of restraint and NMDA treatment were analyzed using the three-ways ANOVA over time (over the four tests) and using the two-ways ANOVA per test. Restraint and NMDA treatment were considered as between subject factors and time as a within subject factor. In the case of significance, Bonferroni corrected t-tests were used. BASAR effects were analyzed using Spearman correlation tests to evaluate the link between the quantitative behavioral scores (latencies to escape from the 50 °C plate in sec, heat avoidance (Δ_28–50 °C_) in sec) and the 3 BASAR levels (0, 1.5, 6 W/kg). Significance was set at *p* < 0.05.

## 3. Results

### 3.1. Effects of the BASAR and of NMDA Injection on Latency to Escape from the Hot Plate

**Figure 2:**
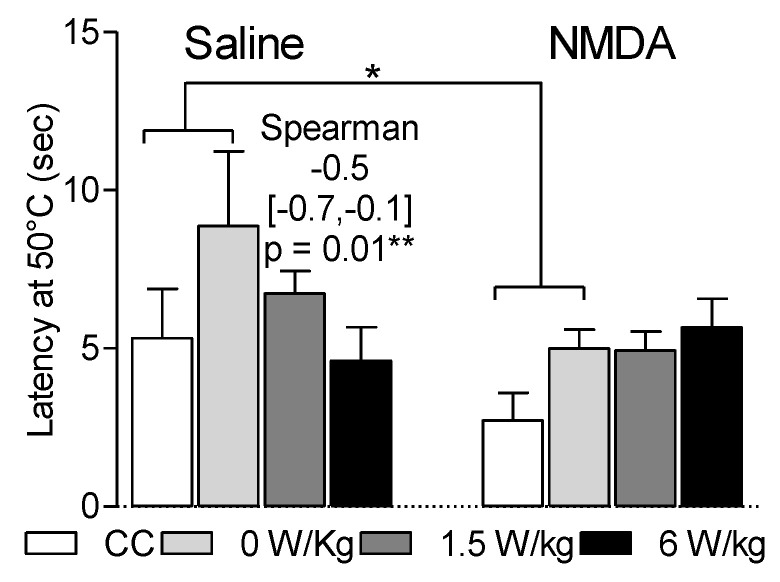
Faster escape from the hot plate in correlation with the BASARs. Sham-exposed and RF-EMF-exposed groups were tested in the two temperatures choice test following i.t. saline or NMDA injection. * *p* < 0.05, escape from the hot plate was faster in the NMDA compared to the saline treated rats. ** *p* = 0.01. Escape from the hot plate was faster in correlation with the BASARs in the saline group but not in the NMDA group. *N* = 6–12 rats/group.

Data reported in Figure 2 presents the latency to escape from the 50 °C plate. Over time, analysis on the four tests indicated the absence of time effect (*p* < 0.05). However, there was an effect of the BASARs and of NMDA treatment on the first test.

In the first test, the latency to escape from the 50 °C plate was not significantly affected by restraint (F1,32 = 3.9, *p* = 0.06). A significant effect may have been hidden because of the variability of the data.

The latency to escape from the 50 °C plate was shorter at 6 W/kg than at 0 W/kg (4.6 ± 1.1 s versus 8.9 ± 2.4 s), i.e., in correlation with the BASARs (Spearman coefficient: −0.5 [−0.7, −0.1], *p* = 0.01). This suggests that at high BASARs (compared to the ICNIRP limits of 2 W/kg SAR), cerebral RF-EMF exposures may quicken heat perception.

NMDA treatment by itself (in the CC and 0 W/kg exposed rats) induced a faster escape from the hot plate compared to the saline treated rats (F1,32 = 4.8, *p* = 0.04, 3.8 ± 0.73 versus 7.1 ± 2.1). Thus, in accordance with the well-known role played by glutamatergic transmission in pain perception, this data suggest that NMDA treatment quickened nociceptive heat perception.

In the NMDA-treated group, RF-EMF exposure did not modify the latency to escape from the hot plate (*p* = 0.8) and there was no NMDA × RF-EMF interaction. Thus, it may suggest that the glutamatergic stimulation rendered RF-EMF ineffective for reducing latency to escape from a hot nociceptive stimulus. Otherwise, the absence of additive effect may be explained if RF-EMF and NMDA quickened nociceptive heat perception through the same biological pathways.

Data observed in the first test were not reproduced through the three subsequent tests. No effect of restraint, NMDA treatment or BASAR was reported on the latencies to escape from the 50 °C plate (Table 1). Data variability may be due to side effects of the repetition of the procedures of NMDA i.t. administrations, restraint, heat nociceptive stimulation and/or RF-EMF exposures.

### 3.2. Dependence of Heat Avoidance on Restraint and BASAR

**Figure 3:**
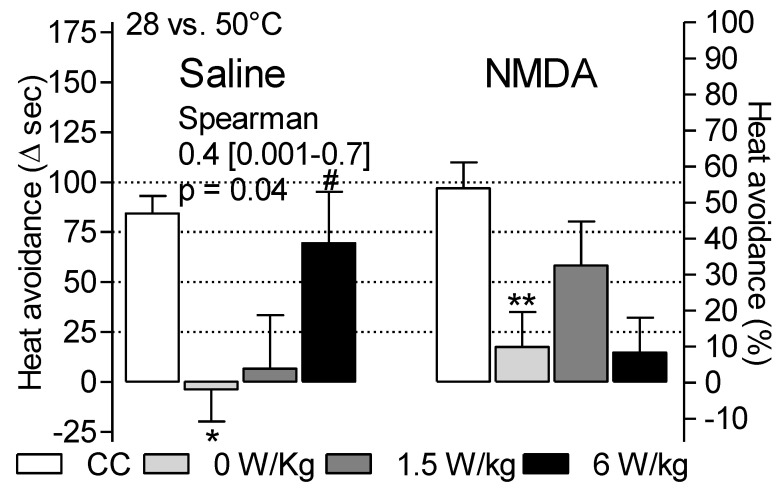
BASAR-dependent increase of heat avoidance (Δ_28–50 °C_ sec) during the first test. Sham-exposed and RF-EMF-exposed groups were tested in the two-temperatures choice test following i.t. saline or NMDA injection. * *p* < 0.05, ** *p* < 0.01 restraint significantly abolished heat avoidance. # *p* = 0.01, heat avoidance increased with the BASARs in the saline treated rats but not in the NMDA-treated rats. *N* = 6–12 rats/group.

Data reported in Figure 3 presents heat avoidance calculated as the delta between the time spent at 28 °C and the time spent at 50 °C (Δ_28–50 °C_). The overall analysis on the four tests indicated a significant time effect after repeated restraints (*p* < 0.05) (Table 2) but BASAR, NMDA and restraint effects only during the first test (*p* < 0.05).

Restraint significantly reduced heat avoidance during the first test (Δ_28–50 °C_ = 92.8 ± 9.0 s in CC versus 6.4 ± 11.8 s in sham rats, F1,24 = 18.6, *p* = 0.0002) but not during the fourth test. It suggested a habituation process to repeated restraints.

During the first test, heat avoidance was increased at 6 W/kg compared with 0 W/kg (Δ_28–50 °C_ = 69.5 ± 25.7 versus −3.6 ± 16.1), i.e., in correlation with the BASARs (Spearman coefficient: 0.4 [0.001–0.7], *p* = 0.04). Preference for the 28 °C side was significant in the 6 W/kg-exposed rats (*p* < 0.05) but not in the sham rats. It suggested that RF-EMF exposures increased heat perception at high BASARs.

NMDA treatment or its interaction by restraint did not modify heat avoidance (respectively, F1,24 = 0.8, *p* = 0.4 and F1,24 = 0.05, *p* = 0.8). This data was not expected as the role played by glutamatergic transmission is well-known in pain perception. Data variability and a lack of statistical power may explain this discrepancy.

Heat avoidance was significantly impacted by the NMDA × RF-EMF exposure interaction (F2,46 = 3.3, *p* = 0.047). In the 1.5 W/kg-exposed rats, Δ_28–50 °C_ reached 58.3 ± 22.0 while it reached only 17.6 ± 17.4 and 14.8 ± 17.4 in the sham and the 6 W/kg-exposed groups. Here, spinal NMDA activation influenced RF-EMF effect with the trend to increase heat sensitivity at 1.5 W/kg and to reduce it at 6 W/kg. Despite the need for further studies, this data suggests that an interaction between NMDA transmission and RF-EMF exposure may occur.

Data observed in the first test were not reproduced through the three subsequent tests. No significant restraint, NMDA treatment or BASAR effect were reported (Table 2). Data variability may be due to side effects of the repetition of the procedures of NMDA i.t. administrations, restraint, heat nociceptive stimulation and/or RF-EMF exposures.

### 3.3. NMDA-Related Increased Locomotor Activity

**Figure 4:**
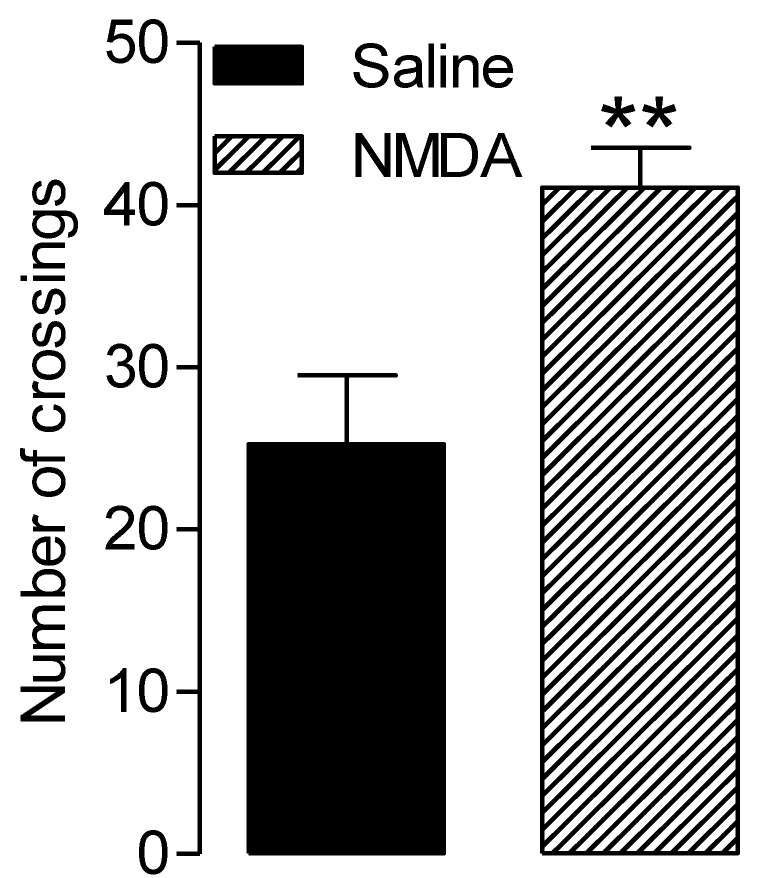
Effect of NMDA on the number of crossings between the two plates in the two temperatures choice test. Sham-exposed and RF-EMF-exposed groups were tested following i.t. saline or NMDA injection. As there was no BASAR or restraint effect, data were pooled for each pharmacological treatment. ** *p* < 0.01, the NMDA-treated rats crossed the plates more frequently compared to the saline treated rats.

Figure 4 presents the total number of crossings between the two plates on the four test sessions. As there was no BASAR or restraint effect, data were pooled for each pharmacological treatment. The number of crossings was significantly higher in the NMDA-treated group compared to the saline-treated group (*p* = 0.004).

This data is in accordance with the hyper locomotor effect of NMDA previously reported in the literature [28]. High locomotor activity may be a confounding factor for interpretation of behavioral responses. For example, it may impact the latency to escape from a nociceptive stimulus.

## 4. Discussion

Here, we hypothesized that high BASAR RF-EMF exposures may impact thermal nociception with a modulatory role played by the NMDA receptor. Our data suggested an increased heat avoidance and a reduced latency to escape from the hot plate in the 6 W/kg group compared to the sham group. This effect was abolished after the NMDA treatment.

Previous studies using subjective scales or reflex responses in the hot plate paradigm suggested contradictory RF-EMF effects on thermal sensitivity [13,15,29,30]. Here, RF-EMF effects on thermal sensitivity were assessed for the first time in an operant paradigm in the rat. This approach allowed objective and quantitative measures of supraspinally-organized responses. A high thermal nociceptive stimulation was induced on the plate at 50 °C. The neutral temperature allowed the rat to avoid the possible heat lesions observed in the inescapable hot plates [31]. BASARs were higher than ICNIRP limits in order to look for hazard effects. Cell phone emissions are usually below and less frequently equivalent to these limits. ICNIRP limits for cell phone RF-EMF to the head are 2 W/kg at the skin and skull levels. The equivalent for the whole rat brain was about 0.5 W/kg BASAR. Here, 6 W/kg and 1.5 W/kg BASAR in the rat were approximatively equivalent to 24 W/kg and 6 W/kg at the skin and skull levels in humans. Numerical and experimental dosimetry were previously performed with precision by Leveque et al. [26]. Rodents were exposed in restraint to the loop antennas. This system mimics cerebral exposures in human brains during a phone call.

Our data suggested that RF-EMF exposure increased heat avoidance at high BASARs. According to our results, the BASAR threshold impacting heat avoidance may be in the range between 6 to 12 times higher than the ICNIRP limits to the brain. The present data affords some support to explain possible subjective symptoms of pain. However, the effects occurred at high but not at environmental BASAR levels. Thus, our results don’t support the hypothesis that RF-EMF environmental exposure could impact public health because of abnormal pain perception effect.

Mechanistically, one may hypothesize that local skin thermal sensitivity was modulated in relationship to whole-body thermal states [32]. Indeed, RF-EMF exposures were previously shown to modify thermal perception or to increase whole temperature in rodents [33,34]. In addition, one may hypothesize that local skin thermal sensitivity was modulated through the chemical mediators influencing the higher-order midbrain regions controlling pain perception. Indeed, some studies suggested that cortisol release, central catecholamine or opioid peptide (endorphin) were influenced by RF-EMF exposures [15,16,17,18,35,36,37,38].

NMDA is the receptor of glutamate, exerting an excitatory transmission in the nervous system. Spinal cord NMDA transmission plays an important role in the control of pain perception. It can be reduced through the central descending pathway and ends up lowering cortical pain discrimination [23]. Here, the stimulation of NMDA transmission in the spinal cord shortened latency to escape from the hot plate. It suggested a faster heat perception. NMDA hyper locomotor effect was in accordance with previous data in intermediate-spinal cats [28].

Here spinal NMDA activation influenced RF-EMF effect with the trend to increase heat sensitivity at 1.5 W/kg and to reduce it at 6 W/kg. The 1.5 W/kg RF-EMF exposure may have modulated spinal pain processes through chemical mediators influencing the higher-order midbrain regions controlling pain perception. Surprisingly, heat escape latency seemed to not be reduced at 1.5 W/kg, maybe because of the hyper locomotor activity induced by NMDA.

NMDA transmission stimulation is known to be followed by a phase of glutamatergic receptor inactivation. During this phase, pain cannot be modified by a subsequent spinal NMDA administration [23,39]. One may hypothesize that 6 W/kg RF-EMF exposure induced a faster glutamatergic receptor inactivation, thus explaining the trend to reduced heat sensitivity.

Restraint is an inescapable stress, causing a peak of corticosterone. Here, five daily restraints reduced heat sensitivity. Accordingly, a previous study indicated that confinement in a RF-EMF restraint exposure chamber elicited restraint-induced analgesia [17]. Stress is often and initially adaptive and elicits anti-nociceptive and acute analgesic mechanisms via the endogenous regulatory systems of nociception [40]. This process promotes the fight or flight response, which enables an organism facing a threat to escape [41,42]. On the contrary, after 20 daily restraints, heat sensitivity returned to normal. It suggested adaptation. Despite the remaining release of stress hormones, stress response evolved to the absence of homeostasis in response to repeated challenges in the environment.

Here, experiments were performed only in males to be consistent with our previous work showing RF-EMF cerebral effects [18,19]. Future experiments dedicated to test sex differences may be justified, as it is not clear whether women present a higher EHS prevalence or are more likely to report their symptoms than men [1].

## 5. Conclusions

The present data affords some support to explain possible subjective symptoms of pain at high, but not at environmental BASAR levels. Our data supports the hypothesis that exacerbated glutamatergic transmissions may influence RF-EMF effects on nociception. Further studies are needed to confirm this data.

## Figures and Tables

**Table 1:** Latencies (sec) to escape from the hot plate in the second, third and fourth tests. Sham-exposed and RF-EMF-exposed groups were tested in the two temperatures choice following i.t. saline or NMDA injections. There was no BASAR, NMDA or restraint effect. *N* = 6–12 rats/group.

		Test 2		Test 3		Test 4	
		Mean (sec)	SE	Mean (sec)	SE	Mean (sec)	SE
Saline	0 W/kg	10.1	2.4	21.0	6.5	6.8	1.9
	1.5 W/kg	7.1	1.7	15.5	5.3	8.0	2.1
	6 W/kg	9.9	3.1	8.8	1.8	7.0	0.9
NMDA	0 W/kg	7.5	1.0	7.3	1.1	10.0	2.1
	1.5 W/kg	8.9	2.6	12.4	2.4	8.1	0.6
	6 W/kg	14.8	4.3	7.1	0.9	6. 6	0.7

**Table 2:** Heat avoidance (Δ_28–50 °C_ sec) on the 2nd, 3rd and 4th tests. Sham-exposed and RF-EMF-exposed groups were tested in the thermal preference in the two-temperatures choice test following i.t. saline or NMDA injection. There was no BASAR, NMDA or restraint effect. * *p* < 0.05 heat avoidance was increased on the fourth test compared to the first test. *N* = 6–12 rats/group.

		Test 2		Test 3		Test 4	
		Mean Δsec	SE	Mean Δsec	SE	Mean Δsec	SE
Saline	0 W/kg	69.0	26.2	29.6	22.3	93.8 *	15.5
	1.5 W/kg	93.3	41.2	34.3	45.9	132.3 *	19.2
	6 W/kg	48.2	22.4	68.9	15.8	86.5 *	16.9
NMDA	0 W/kg	52.6	24.9	81.3	9.4	51.5	26.8
	1.5 W/kg	51.5	40.8	23.0	37.7	116.5 *	17.6
	6 W/kg	37.0	26.0	69.1	22.3	91.3 *	21.5
